# Fatal Case of Nosocomial *Legionella pneumophila* Pneumonia, Spain, 2018

**DOI:** 10.3201/eid2511.181069

**Published:** 2019-11

**Authors:** Diego Vicente, Jose M. Marimón, Itziar Lanzeta, Tania Martin, Gustavo Cilla

**Affiliations:** Universidad del País Vasco, País Vasco, Spain (D. Vicente);; Hospital Donostia-Instituto Biodonostia, San Sebastian, Spain (D. Vicente, J.M. Marimón, I. Lanzeta, T. Martin);; Centro de Investigación Biomédica en Red Enfermedades Respiratorias, Madrid, Spain (D. Vicente, J.M. Marimón, G. Cilla)

**Keywords:** *Legionella pneumophila*, urinary antigen test, UAT, pneumonia, respiratory infections, bacterial infections, bacteria, Legionnaires’ disease, nosocomial infections, Spain

## Abstract

A nosocomial case of *Legionella pneumophila* pneumonia likely caused by a serogroup 3 strain was detected by a urinary antigen test in Spain in 2018. Although *Legionella* bacteria could not be isolated from respiratory samples, molecular methods implicated the sink faucet of the patient’s room as the probable infection source.

Legionnaires’ disease (LD) is a severe form of pneumonia caused primarily by the inhalation of *Legionella* spp. bacteria from colonized natural and artificial water systems. The annual incidence of LD in developed countries is ≈1 case/100,000 inhabitants and has been increasing in recent years (*1*,*2*). This increase is attributed in part to the widespread use of the urinary antigen test (UAT) as a diagnostic method. UAT is a fast and simple method, with good sensitivity and specificity to confirm the clinical suspicion of LD; however, most commercial tests only detect *Legionella pneumophila* serogroup 1. In many countries, UAT is used as the only LD diagnostic method. This strategy leads to the loss of detection of episodes caused by non–serogroup 1 *L. pneumophila*, including *L. pneumophila* serogroups 2–14, and other *Legionella* species (*1*,*2*).

We describe an episode of nosocomial *L. pneumophila* pneumonia probably caused by a serogroup 3 strain, which was diagnosed by UAT and genomic detection of *Legionella* DNA in respiratory samples. The episode was linked to the detection of the causative agent in the water of the sink of the patient’s room.

## The Study

In February 2018, a 66-year-old man sought care at Hospital Donostia-Instituto Biodonostia (San Sebastian, Spain) because of progressive loss of strength, accompanied by dysarthria and altered state of consciousness. A cranial computed tomography scan performed at hospital admission showed a deep intraparenchymal hematoma and a substantial surrounding edema. After a 1-month hospitalization in the neurology department, the patient was transferred to the long-stay unit of the internal medicine department, where he occupied the same room until the end of the episode. During this period, he was treated with high doses of dexamethasone to reduce the cerebral edema and different cycles of antibiotics (piperacillin/tazobactam and ceftriaxone) because of the presence of abundant respiratory secretions.

In April 2018, the patient had acute worsening of respiratory function, requiring high oxygen flow rates and mechanical ventilation. A chest radiograph showed the appearance of bilateral pulmonary infiltrates, and we observed elevated sepsis-associated markers in the blood analysis.

The patient was given a presumptive diagnosis of nosocomial pneumonia. We obtained a urine sample, 2 blood cultures, and 2 respiratory samples (sputum and tracheal aspirate) for microbiologic analysis. Blood cultures were negative. Results of a fluorescent immunoassay (Sofia *Legionella* FIA, https://www.quidel.com) detected *L. pneumophila* antigen in the urine, a result that was confirmed in a second sample obtained 12 hours later. Both urine samples had a negative result when tested with the Alere BinaxNOW *Legionella* Antigen Test Kit (Fisher Scientific, https://www.fishersci.com), which only detects *L. pneumophila* serogroup 1 (*3*). In the microbiologic culture of respiratory samples, we observed a scanty growth of saprophytic bacteria, but a culture on selective media *Legionella* (BCYE agar) was negative. The multiplex PCR for detection of *Legionella* spp., *Chlamydia pneumoniae*, and *Mycoplasma pneumoniae* (BioGX, https://biogx.com) performed on the BD MAX System (https://www.bd.com) was positive for *Legionella* spp., both in the sputum and in the tracheal aspirate. The patient received levofloxacin but died 48 hours later.

After establishing the diagnosis of LD, we conducted an investigation to determine the origin of the episode and monitored the appearance of more cases. No episodes of *Legionella* spp. pneumonia were detected among patients admitted to the same unit during the previous month and during the month after the episode. We obtained samples of water from 23 different points of the internal medicine department unit where the patient had stayed, including his room’s sink faucet and shower as well as another 5 rooms, an office, spillways, and refrigeration equipment.

Non–serogroup 1 *L. pneumophila* (i.e., serotyped 2–14 in our microbiology department) was isolated in glycine, vancomycin, polymyxin, cycloheximide agar plates from the sink faucet of the patient’s room (1,250 cfu/L) and from the sink faucet (275 cfu/L) and shower faucet (1,250 cfu/L) of the contiguous room. Disinfection of the affected facilities through thermal shock was performed, and the disappearance of *Legionella* was verified by using the same methods described. Monoclonal antibody subgrouping conducted at Spain’s National Center for Microbiology identified *Legionella* isolates from these 3 environmental samples as serogroup 3. No more *L. pneumophila* was detected in any of the other 20 water samples we analyzed.

We performed sequence-based typing on DNA extracted from the sputum and the tracheal aspirate of the patient and from the 3 environmental isolates. We sequenced and amplified fragments of 7 genes in accordance a with protocol established by the European Working Group for Legionella Infections (EWGLI) (*4*), which includes the use of specific primers for the *neuA* homologue allele *neuAh* (*5*). We applied a nested PCR–derived sequence based typing method directly to DNA extracted from respiratory samples (*6*) and assigned allele and sequence type by using the online EWGLI SBT Database (*7*). The *Legionella* spp. detected in the 2 respiratory samples of the patient, as well as in the water of the patient’s and a contiguous room, were identified as sequence type 1341.

## Conclusions

Most LD episodes reported worldwide are attributed to *L. pneumophila* serogroup 1. Nevertheless, several studies suggest that episodes caused by *L. pneumophila* other than serogroup 1 might be underestimated because the main current method used for microbiologic diagnosis of LD is the UAT, which in most commercial test kits is limited to the detection of *L. pneumophila* serogroup 1 (*8*–*10*). The analysis of isolates from patients with *Legionella* pneumonia, both in Europe and the United States, shows that up to 20% were caused by *L. pneumophila* serogroups 2–14 or *Legionella* other than *L. pneumophila* (*1*,*2*,*11*), and this rate is higher among hospital-associated cases (*12*). However, *Legionella* culture is rarely used as a routine diagnostic method (*1*,*2*) because of the low efficiency and difficulties in isolation even if the bacterial DNA is present in the sample, as in the case we have described.

In our study, antigen detection in urine was performed by using a method able to detect, in addition to *L. pneumophila* serogroup 1, lipopolysaccharide of most *L. pneumophila* serogroups, including serogroup 3, although with a higher limit of detection (*3*). In addition, a nucleic acid amplification technique that enables detection of all *Legionella* species was used in respiratory samples, and secondarily, helped indicate an epidemiologic link with the infectious source without microbiologic culture of the causative strain. Although strains of *L. pneumophila* with identical sequence type can be of different serogroups, our results strongly suggest that the *L. pneumophila* serogroup 3 ST1341 strain isolated from the water in the sink faucet of the patient’s room was the source of the nosocomial pneumonia episode. The *L. pneumophila* serogroup 3 ST1341, probably responsible for the nosocomial pneumonia episode we describe, was also reported in the EWGLI SBT Database in 3 LD episodes (2 in Germany and 1 in Catalonia, Spain) (*7*) and in an environmental sample in Greece (*8*). The strain does not seem to be a frequent genotype, although the case we have described supports the potential of nearly all *Legionella* genotypes to cause fatal infection.

Even though *L. pneumophila* serogroup 1 is responsible for most LD episodes, it is advisable to have techniques to detect other serogroups and species of *Legionella*, especially in the hospital environment, where the presence of non–serogroup 1 *L. pneumophila* is frequent (*8*,*12*) and a high number of susceptible immunosuppressed patients are present.
